# Perceived Susceptibility to and Seriousness of COVID-19: Associations of Risk Perceptions with Changes in Smoking Behavior

**DOI:** 10.3390/ijerph18147621

**Published:** 2021-07-17

**Authors:** Erin A. Vogel, Lisa Henriksen, Nina C. Schleicher, Judith J. Prochaska

**Affiliations:** Stanford Prevention Research Center, Department of Medicine, Stanford University, Medical School Office Building, X316, 1265 Welch Road, Stanford, CA 94305, USA; eavogel@stanford.edu (E.A.V.); lhenriksen@stanford.edu (L.H.); schleicher@stanford.edu (N.C.S.)

**Keywords:** smoking, tobacco, cigarettes, COVID-19, Health Belief Model

## Abstract

During the COVID-19 pandemic, studies have documented increased and decreased cigarette smoking among adults. Individual differences in the perceived susceptibility and seriousness of the virus, for people who smoke in general and for oneself personally, may relate to changes in smoking. Using the Health Belief Model (HBM) as a theoretical framework, we examined associations with self-reported increasing and decreasing smoking a lot during the COVID-19 stay-at-home period. Adults in 30 large U.S. cities who smoked cigarettes daily completed an online survey between 14 July and 30 November 2020. The analytic sample (N = 2768) was 54.0% male and 68.3% white with 23.7% reporting increasing and 11.3% decreasing smoking (6% reported both). Younger age, a diagnosis of COVID-19, and greater pandemic-related stress were associated with greater odds of both increased and decreased smoking. Increased smoking also was associated with heavier nicotine dependence, greater desire to quit, and greater perceived susceptibility and lower perceived seriousness of COVID-19 for people who smoke, while pandemic-related job-loss, lower nicotine dependence, and greater self-efficacy were associated with decreased smoking. Among respondents who had not contracted COVID-19 (*n* = 2418), correlates were similar with the addition of greater perceived personal susceptibility to COVID-19 associated with both increased and decreased smoking, while greater perceived personal seriousness of COVID-19 was associated with increased smoking. Findings for risk perceptions were largely in directions that contradict the HBM. Circumstances surrounding behavior change during the pandemic are complex and may be especially complex for nicotine addiction.

## 1. Introduction

The COVID-19 pandemic has resulted in unprecedented global mortality and morbidity and represents a major source of stress and disruption to daily life. As of June 2021, the World Health Organization has reported over 177 million confirmed cases of COVID-19, including nearly 4 million deaths [1]. People who smoke cigarettes may be particularly vulnerable to contracting COVID-19 and developing serious complications from the virus. Smoking increases risk for serious respiratory illness and inflammation following viral infection, which may worsen COVID-19 outcomes [2]. Indeed, a recent review concluded that severe COVID-19 symptoms are more likely among people who smoke than those who do not [3]. In the United States, the Centers for Disease Control and Prevention disseminated public guidance stating that current and former smoking can increase risk for severe COVID-19 [4]. On the other hand, there is biological plausibility for a protective role of nicotine against severe COVID-19 [5]. Observational studies in China [6] and France [7] reported a lower-than-expected prevalence of smoking among people hospitalized with COVID-19. After preprints suggesting the potential protective effects of tobacco were circulated online, positive sentiments around smoking and COVID-19 increased on Twitter [8]. Receiving mixed messages, people who smoke may be uncertain how smoking affects their COVID-19 risk. Such uncertainty could undermine efforts to quit smoking.

The COVID-19 pandemic has had different effects on smoking behavior. A cross-sectional survey of U.S. adults who use cigarettes and/or e-cigarettes found that compared to pre-pandemic, 28.3% of participants reported decreasing their smoking, while 30.3% reported increasing [9]. In a similar web-based survey of Belgian adults, including adults who did not smoke prior to the pandemic, 7.4% of the full sample reported increased smoking and 2.5% reported decreased smoking. Increased smoking was more likely among younger adults, those who lived alone, those with less education, and those without employment [10]. In a U.S. study, college students who smoked prior to pandemic-related campus closure reported significantly fewer smoking days during campus closure, and more than one quarter of them paused their tobacco use entirely [11]. In a small study of older adults, 27.6% reported increased and 20.7% decreased smoking [12]. Data from the Nielsen National Consumer Panel, representative of U.S. consumers, showed a 13.2% increase in tobacco sales during 1 April–30 June 2020, compared to the same dates in 2019 [13]. Across adults of varying ages in the U.S. and Belgium, the reported reasons for decreasing smoking include spending more time around one’s children [14], spending less time in social situations that promote smoking [12,14,15,16], difficulty obtaining or affording cigarettes [12,15,16], and health concerns [12,14,15,16]. The reported reasons for increased smoking include increased stress [12,14,15,16], boredom [10,12,14,15], less social support for quitting smoking [15], and having greater flexibility in one’s schedule [14,16].

Individual differences in risk perceptions may be related to smoking behavior during the pandemic. The Health Belief Model (HBM) was developed in the 1950s by social psychologists working in the U.S. Public Health Service to explain why people did not participate in a free and accessible tuberculosis screening program. According to the HBM, individuals’ likeliness to engage in a behavior is largely determined by their perceived susceptibility to the health threat and perceived seriousness of the health threat [17]. Over time, the application of the HBM broadened to interpret and understand individual differences in a wide variety of preventive health practices, such as colorectal cancer screening [18] and the use of face masks to prevent respiratory disease prior to COVID-19 [19]. Therefore, the model may offer insight into people’s smoking behavior during the COVID-19 pandemic. Beliefs that smoking would increase one’s risk of getting COVID-19 (i.e., high perceived susceptibility) and/or would increase the severity of one’s experience of COVID-19 (i.e., high perceived seriousness) may lead to decreased smoking during the pandemic in an attempt to mitigate risk. Other constructs of the HBM are modifying variables in the form of demographic and psychological characteristics, perceived benefits and barriers of taking preventive action, internal and external cues to action [17], and self-efficacy (i.e., a person’s belief in their ability to engage in the behavior of interest) [20]. Changes in smoking may also affect risk perceptions. People who increase their smoking during the pandemic may downplay the impact their smoking may have on their COVID-19 risks.

During the COVID-19 pandemic, most studies of the HBM focused on behavioral intentions (e.g., vaccination, use of a contact-tracing app), and adherence to COVID-19 mitigation behaviors (e.g., handwashing, social distancing), with mixed results. Perceived susceptibility and seriousness were positively associated with intention to obtain a vaccination in three studies [21,22,23], but not in two other studies [24,25]. In a nationally representative online survey of U.S. adults conducted in August 2020, greater perceived seriousness was associated with greater odds of intending to receive the COVID-19 vaccine, while perceived susceptibility was not [26]. Among Belgian adults, perceived susceptibility to and seriousness of COVID-19 were not associated with intention to use a contact-tracing app [27]. Similarly, in a survey of adults in Macao, China, perceived susceptibility was not significantly related to adherence to COVID-19 mitigation behaviors. Perceived seriousness was associated with only one of six behaviors (i.e., proper toilet flushing) [28]. Likewise, a survey of employed adults in Ethiopia’s capital city found that susceptibility to and severity of COVID-19 were not related to COVID-19 prevention practices [29]. Specific to smoking, a survey of adults in Ohio found that perceived susceptibility to severe COVID-19 infection was associated with greater desire to quit smoking [30]. Decreasing one’s smoking primarily benefits oneself and one’s household members, while other pandemic-relevant behaviors (e.g., contact tracing, prevention practices) are frequently described as benefiting one’s broader community. Susceptibility and seriousness may be more personally relevant for smoking than for other pandemic-related behaviors. Moreover, several previous studies measured behavioral intentions exclusively, and did not measure behaviors. Few studies have focused on changes in behaviors in which people are already engaging, such as increased or decreased smoking. A greater understanding of how individual perceptions of COVID-19 susceptibility and seriousness relate to tobacco use during the pandemic is needed.

The present study is an online survey of adults in 30 U.S. cities who smoke cigarettes daily, conducted July through November 2020, when COVID-19 infections and hospitalizations were rising in most parts of the U.S. Analyses examined associations between perceived susceptibility and perceived seriousness of COVID-19 with reported changes in smoking behavior during COVID-19 stay-at-home restrictions. Because smoking behavior could vary during the stay-at-home period, we measured increased and decreased smoking separately. We assessed COVID-19 threat perceptions (susceptibility and seriousness) for oneself (personally) and for people who smoke (generally). Based on the tenets of the HBM, we hypothesized that greater perceived susceptibility to and seriousness of COVID-19 would be negatively associated with increased smoking and positively associated with decreased smoking during the pandemic. Our models adjusted for relevant modifying variables (e.g., respondent gender, age, race/ethnicity, heaviness of smoking, pandemic-related job and income loss, desire to quit smoking, and COVID-19 diagnosis) and examined the association of self-efficacy with changes in smoking.

## 2. Methods

### 2.1. Participants and Procedure

The participants were adults who smoked cigarettes daily, resided in one of 30 large U.S. cities (see Appendix A Table A1), and were participating in the Big City online survey as part of the Advancing Science and Practice in the Retail Environment (ASPiRE) multi-institutional consortium (grant #P01-CA225597). The eligibility criteria for survey participation were age 21–59, living in a zip code contained entirely or mostly within one of the 30 cities, not intending to relocate in the next two years, English literate, and self-reported smoking cigarettes daily (i.e., 100+ lifetime cigarettes and at least 1 cigarette per day in the past 7 days). Informed consent was obtained from all participants. Recruitment was conducted via Qualtrics Research Services and supplemented with Craigslist advertisements. Qualtrics participants were compensated $11–17 in e-rewards points. E-rewards points are exchangeable for gift cards or bank transfers, with the exact value dependent on the incentive selected. Craigslist participants in a subset of cities with low recruitment via Qualtrics (see Appendix A Table A1) were compensated $15. Outcome measures and primary predictor variables presented are from Wave 2 of a longitudinal survey conducted 14 July 2020–30 November 2020, during the COVID-19 pandemic. At Wave 2, participants were sampled with replacement: 78.9% (*n* = 2185) of the analytic sample were new respondents and 21.1% (*n* = 583) were returning respondents.

### 2.2. Measures

#### 2.2.1. Perceived Susceptibility and Seriousness of COVID-19

Personal susceptibility was assessed with, “How likely do you think it is that you will be diagnosed with COVID-19 within the next year?” (1 = not at all likely; 5 = very likely). Personal seriousness was measured with, “How serious do you think infection with COVID-19 would be to your health?” (1 = not at all serious, 5 = very serious). Only participants (88.4%, *n* = 2447) who had not had COVID-19 (i.e., no self-reported positive test or medical diagnosis) reported personal susceptibility and seriousness. Among all participants, two items assessed susceptibility to and seriousness of COVID-19, respectively, for people who smoke: “Smoking cigarettes _____ the risk of getting COVID-19” and “Smoking cigarettes ______ the risk of dying from COVID-19” (1 = greatly increases; 2 = increases; 3 = does not change; 4 = decreases; 5 = greatly decreases).

#### 2.2.2. Changes in Smoking

In two separate questions, participants reported the extent to which they smoked more than usual and smoked less than usual during the COVID-19 pandemic stay-at-home period in their city (no; yes, a little; yes, a lot; don’t know). Instructions defined this period as a time in which “many U.S. residents were asked to limit their activities and stay at home” and noted that for most people, these restrictions were in place for the entire month of April 2020 but may have started earlier and/or ended later in the respondent’s city.

#### 2.2.3. Pandemic Effects

Participants reported whether they suspected having had COVID-19 (yes, with positive test; yes, medical diagnosis, but no test; yes, have had some possible symptoms, but no diagnosis by a doctor; no symptoms or signs). Participants with a positive test or medical diagnosis were considered to have had COVID-19. Participants reported whether they lost their job permanently during the pandemic (yes/no) and changes in income as a result of the pandemic (decreased; did not change; increased; don’t know). The four-item Perceived Stress Scale [31] (e.g., “How often did you feel confident about your ability to handle your personal problems?”) measured stress during the stay-at-home period (0 = never, 4 = very often). 

#### 2.2.4. Smoking Characteristics

The two-item Heaviness of Smoking Index [32], scored on a 0–6 scale summing the two items, measured cigarettes per day (0 = 10 or fewer, 1 = 11–20, 2 = 21–30, 3 = 31 or more) and time to first cigarette upon wakening (3 = within 5 min, 2 = 6–30 min, 1 = 31–60 min, 0 = after 60 min). A single item assessed self-efficacy for quitting smoking (i.e., how successful one would be at quitting smoking now) rated from 1 = not successful to 10 = entirely successful. Current desire to quit smoking was reported as 1 = no desire to quit to 10 = full desire to quit. All items were assessed at Wave 2 for all participants, except time to first cigarette, which was assessed only at a participant’s first survey wave (i.e., Wave 1 for returning participants, Wave 2 for new participants).

#### 2.2.5. Sociodemographic Characteristics

Participants reported their age, gender (male, female, transgender, gender non-conforming), race (White/Caucasian, Black or African American, Asian, Pacific Islander/Native Hawaiian, American Indian/Alaska Native, not known, other), and Hispanic/Latinx ethnicity (yes/no). Participants provided demographic data at their baseline wave of survey completion (Wave 1 or Wave 2). Due to small sample sizes, some demographic groups were combined in analyses. Participant gender was categorized as “man” (male) or “womxn” (all other genders). Race and ethnicity were combined into non-Hispanic white or other race/ethnicity; “not known” was treated as missing data. 

## 3. Analyses

Based on the observed distributions, personal susceptibility and seriousness were categorized as low (1–2), moderate (3), or high (4–5). General susceptibility and seriousness were categorized as low (smoking greatly decreases, decreases, or does not change risk) or high (smoking greatly increases or increases risk). Chi-square tests examined associations between personal and general susceptibility and seriousness measures. Participants who reported increasing (decreasing) smoking “a lot” were categorized as “increasing” (“decreasing”). All other responses (i.e., increasing/decreasing “a little,” “not at all,” or “don’t know”) were categorized as not increasing (decreasing).

GEE models, adjusted for covariates and the clustering of participants within 30 cities, examined the association of changes in smoking during the stay-at-home period with perceived susceptibility and seriousness of COVID-19. With the full sample, two GEE models examined the likelihood of increased smoking and decreased smoking as a function of general COVID-19 susceptibility and seriousness for people who smoke. Two additional GEE models, restricted to participants who had not gotten COVID-19, examined the likelihood of increased smoking and decreased smoking as a function of both personal and general susceptibility and seriousness of COVID-19. All four GEE models adjusted for age (collapsed into 4 categories: 21–30, 31–40, 41–50, 51–59), gender (man vs. womxn), race/ethnicity (non-Hispanic White vs. other race/ethnicity), job loss (vs. no job loss), income loss (vs. no income loss or not known), time coded as weeks elapsed since the survey was first fielded (14 July 2020), stress during COVID-19, heaviness of smoking (an indicator of nicotine dependence), self-efficacy for quitting smoking, and desire to quit smoking. The analyses were conducted using IBM SPSS Statistics 27 (IBM Corp., Armonk, NY, USA).

## 4. Results

### 4.1. Participant Characteristics

Participants in the analytic sample were those who provided complete data on increased smoking, decreased smoking, general susceptibility, general seriousness, and all covariates. Among participants who had not had COVID-19, complete data on personal susceptibility and personal seriousness were also required for inclusion in the analytic sample. Of the 3435 participants who completed the survey, 2768 were retained in analyses.

Participant characteristics are presented in Table 1. The analytic sample (N = 2768) was 54.0% male (45.5% female, 0.5% gender minority). The racial makeup of the sample was 68.3% white, 20.6% Black, 4.2% Asian, 3.5% multiracial, and 3.4% other race. Additionally, 13.4% of participants were Hispanic/Latinx. The largest age group was 31–40 years old (41.5%). A substantial proportion of participants reported losing income (40.1%) or a job (21.3%) during the COVID-19 pandemic. Participants reported moderate stress during the stay-at-home period (M = 2.14, SD = 0.63, minimum = 0, maximum = 4). Heaviness of smoking reflected low to moderate nicotine dependence on average (M = 2.53, SD = 1.31, minimum = 0, maximum = 6). The sample’s self-efficacy for quitting smoking (M = 5.45, SD = 2.77) and desire to quit smoking (M = 6.31, SD = 2.69) averaged slightly above the midpoint of the scale (minimum = 1, maximum = 10).

Most participants reported low (60.9%) to moderate (25.6%) personal susceptibility to COVID-19, but moderate (25.6%) to high (48.1%) personal seriousness of COVID-19. About half reported that smoking increases susceptibility to (53.8%) and seriousness of (62.3%) COVID-19. Crosstabulations between personal and general susceptibility and seriousness measures are presented in Table 2. Although the constructs were strongly related, they appeared distinct. For example, 14.8% of participants reported that smoking decreases or does not change susceptibility to COVID-19 but increases seriousness of COVID-19. Likewise, 13.8% reported low general seriousness, but high personal seriousness. Nearly a quarter of participants (29.0%) reported changing their smoking a lot during the stay-at-home period: 23.7% increased smoking, 11.3% decreased smoking. According to a crosstabulation, 6.0% reported both increasing and decreasing.

### 4.2. Changes in Smoking

Table 3 presents the models of changes in smoking during the COVID-19 stay-at-home period as a function of perceptions of general susceptibility and seriousness of COVID-19 evaluated with the full sample (i.e., including participants who got COVID-19), adjusting for covariates and clustering of participants within cities (For the full sample, intraclass correlation coefficients were 0.02 for increased smoking and 0.05 for decreased smoking). Respondents who reported having had COVID-19 were significantly more likely to have altered their smoking behavior by increasing, decreasing, or both increasing and decreasing their smoking during the stay-at-home period. Perceiving high (vs. low) general susceptibility to COVID-19 for people who smoke was associated with significantly greater likelihood of increased smoking but was not significantly associated with decreased smoking. Perceiving high general seriousness of COVID-19 for people who smoke was associated with significantly lower likelihood of increased smoking but was not significantly associated with decreased smoking. Additionally, increased smoking during the stay-at-home period was significantly associated with younger age. Specifically, participants aged 41–50 and 51–59 were significantly less likely to increase their smoking than those aged 21–30. Increased smoking was also significantly associated with greater stress during the pandemic, greater nicotine dependence, and greater desire to quit smoking. Decreased smoking during the stay-at-home period was significantly associated with younger age (i.e., age 21–30), the male gender, job loss during the pandemic, greater stress during the pandemic, lower nicotine dependence, and greater self-efficacy for quitting smoking. 

For the 2418 participants who did not report COVID-19 and who completed measures of general and personal susceptibility and seriousness, Table 4 summarizes models of reported changes in smoking during the stay-at-home period as a function of personal susceptibility and seriousness, general susceptibility and seriousness, and covariates, adjusting for clustering of participants within cities. In two separate models, perceiving high personal susceptibility to COVID-19 was associated with a significantly greater likelihood of increasing smoking and/or decreasing smoking. Moderate personal susceptibility was not associated with changes in smoking. Perceiving high personal seriousness and high general susceptibility were significantly associated with greater likelihood of increased smoking. Moderate personal seriousness was not associated with changes in smoking. Perceiving high general seriousness was associated with significantly lower likelihood of increased and decreased smoking. Associations of increased and decreased smoking with covariates were similar to the associations identified in the full sample, except that greater self-efficacy for quitting smoking was associated with a lower likelihood of increased smoking in the subsample that did not report having had COVID-19.

## 5. Discussion

In a convenience sample of U.S. adults who smoked cigarettes daily and lived in one of 30 cities, perceived susceptibility to and seriousness of COVID-19 for oneself personally and for people who smoke generally were significantly associated with self-reported changes in smoking behavior during the stay-at-home period of the COVID-19 pandemic. The direction of associations varied, such that increasing smoking during the stay-at-home period was associated with significantly greater perceptions of COVID-19 personal susceptibility, personal seriousness, and general susceptibility, but with lower perceptions of COVID-19 general seriousness. Decreasing smoking was significantly associated with greater perceptions of COVID-19 personal susceptibility and with lower perceptions of COVID-19 general seriousness. When participants who had had COVID-19 were included in the analysis, however, perceived general seriousness was no longer significantly associated with decreased smoking. 

The measures of personal and general susceptibility and seriousness were significantly related to one another, suggesting that viewing COVID-19 as a threat was reflected in all four measures of perceived risk. However, a substantial proportion of participants (26.3%) perceived low-to-moderate personal seriousness, yet high general seriousness, as well as low-to-moderate personal susceptibility, yet high general susceptibility (40.9%). Despite their own smoking, many participants seemed to believe that while smoking increases risk for COVID-19, they were at low risk. This finding may have been attributable, in part, to measurement. Participants reported their personal risk perceptions in terms of absolute risk and general risk perceptions in terms of relative risk (i.e., relative to non-smoking). Alternatively, this finding may reflect comparative optimism (i.e., the robust belief that oneself will fare better than most people). Comparative optimism is driven by both motivated (e.g., self-enhancement, anxiety reduction) and nonmotivated processes (e.g., having more information about oneself than about others, having difficulty judging the risk of a large reference group such as “people who smoke”) [33]. It is also noteworthy that among participants who had not had COVID-19, perceived personal seriousness of COVID-19 was greater than perceived personal susceptibility. In other words, participants believed that they were not highly likely to contract COVID-19, but that their illness would be serious if they did contract it. Belief in the seriousness of COVID-19 may have prompted mitigation measures that decreased the participants’ likelihood of contracting COVID-19 (e.g., social distancing, hand hygiene) and therefore accurately decreased their perceived susceptibility to the illness. Indeed, only 11.6% of the sample reported having contracted COVID-19 by mid-2020, suggesting that many participants may have indeed been at low risk. 

Based on the HBM, we hypothesized that participants with greater perceptions of personal and general susceptibility and seriousness would be less likely to increase their smoking and more likely to decrease their smoking during the COVID-19 stay-at-home period. Consistent with the HBM and our hypotheses, participants who did not believe that smoking increased the risk of getting seriously ill from COVID-19 (i.e., low general seriousness) were more likely to increase their smoking during the stay-at-home period. Participants who believed that they were at high risk of contracting COVID-19 (i.e., high personal susceptibility) were more likely to decrease their smoking. However, other associations for the measures of COVID-19 threat perceptions were not in the expected direction. 

Importantly, the HBM was developed to predict volitional behaviors, such as obtaining a tuberculosis test or a vaccine. Rational choice theories, like the HBM, rely on assumptions that people are motivated chiefly by self-interest and that their preferences are fairly stable and based upon their judgments of available information. Such assumptions of choice discount the role of addiction [34]. In adults who smoke cigarettes daily, behavior change is likely complicated by nicotine dependence. Individuals who want to quit or reduce smoking in order to reduce their COVID-19 risk may face difficulty doing so. In this study, participants with greater nicotine dependence, as measured by the Heaviness of Smoking Index, were significantly more likely to increase their smoking and less likely to decrease their smoking during the stay-at-home period. In contrast, those with greater self-efficacy, or belief in their ability to quit smoking, were less likely to increase their smoking and more likely to decrease smoking during the stay-at-home period. Unexpectedly, a greater desire to quit was associated with greater likelihood of increasing smoking. While income loss was not significantly associated with changes in smoking, job loss was associated with decreased smoking. Changes in daily routines following job loss (e.g., more unscheduled time, more time spent with family) may have disrupted the participants’ typical smoking patterns. Taken together, results suggest a complicated relationship between nicotine dependence, thoughts about abstinence, and behavior change. Nicotine dependence, which may serve as a modifying variable in the HBM framework, may hamper efforts to change behavior for health protection despite a desire to do so. 

In the context of nicotine dependence, rational choice is further impeded by the tobacco-saturated media and retail environments in which people live. Even during the COVID-19 stay-at-home period, many tobacco specialty shops remained open, some in direct opposition of state orders mandating them to close [35]. Prior criticisms of the HBM have noted the weak effects of tobacco prevention interventions that target rational decision-making processes, as well as the potential for the misuse of such rational choice theories by the tobacco industry in litigation, namely to blame tobacco’s harms on individuals who use it [34]. Our findings of increased smoking among those who perceived greater COVID-19 susceptibility and personal seriousness suggest something other than a rational decision-making process. That measures of nicotine dependence, stress, and self-efficacy also related to increased smoking provides support that patterns of smoking behavior are not well-described by rational choice theories like the HBM.

The unique circumstances of the pandemic, including stress, isolation, and economic hardship, also bring complexity. The COVID-19 stay-at-home period was marked by significant uncertainty and rapid changes in available information, making it difficult for people to accurately assess threat. Additionally, the COVID-19 pandemic has produced unprecedented disruption in every facet of daily life. Participants who lost their job during the pandemic were significantly more likely to decrease their smoking, suggesting that they may have tried to save money by purchasing fewer cigarettes during financial hardship. Perceived stress from the pandemic was significantly associated with both increased smoking and decreased smoking. Prior research has also documented both increased and decreased smoking during the pandemic [9,10,12], with people reporting a variety of reasons for changes in their smoking, such as disrupted schedules and social situations [12,14,15,16], financial and logistical constraints [12,15,16], and stress [12,14,15,16]. While health concerns of smoking and COVID-19 contributed to decreased smoking [12,14,15,16], other factors were also influential. Other studies using the HBM to predict behavior and behavioral intentions during the pandemic [21,22,23,24,25,26,27,28,29,30] have also produced mixed results. While existing behavior change theories provide a foundation for understanding health behaviors, they may be less predictive of behavior during the unique time of the COVID-19 pandemic.

### 5.1. Limitations and Future Directions

In the current secondary analysis, we were unable to examine all constructs of the HBM. Perceived benefits and barriers to behavior change, which were not assessed, are often stronger predictors of behavior than susceptibility and seriousness [36] and may have provided insight into changes in smoking behavior. Future research should examine the role of all HBM constructs in pandemic-related changes in smoking behavior. The sample, while large and geographically and demographically diverse, is not representative of all U.S. adults smoking daily in urban areas. Individuals who lost jobs or income may have been more likely to participate in research studies for compensation, due to financial concerns and increased free time.

Lastly, items of interest (e.g., current risk perceptions and smoking behavior during the stay-at-home period) were focused on temporally distinct time periods. If COVID-19 risk perceptions changed during the pandemic, smoking behavior during the stay-at-home period would have preceded measured risk perceptions, and it is plausible that participants who changed their smoking subsequently changed their risk perceptions. For example, participants who increased their smoking during the stay-at-home period may have increased their perceived personal susceptibility and seriousness to COVID-19 due to their increased smoking. Additionally, they may have viewed smoking as increasing susceptibility because their heavier smoking raised the personal salience of a potential link between smoking and COVID-19 risk. Other variables in the model, such as heaviness of smoking, which is dependent on the number of cigarettes smoked per day, may have been influenced by changes in smoking behavior during the stay-at-home period. Regardless of the temporal order of changes in smoking and COVID-19 risk perceptions, some results contradict the HBM. Relationships may be bidirectional, and this cross-sectional study did not enable causal inference. Future research could use methodology more sensitive to short-term changes in perceptions and behavior, such as ecological momentary assessment, to further examine the relationships between risk perceptions and smoking behavior.

### 5.2. Conclusions

The COVID-19 pandemic created a multitude of stressors and concerns and changes in daily schedules that may affect smoking behavior. Among U.S. adults smoking daily in 30 U.S. cities, the threat of COVID-19, perceived personally and for people who smoke generally, was associated with changes in smoking behavior, however, largely in directions that contradict the HBM. The circumstances surrounding behavior change during the pandemic are complex and may be especially complex for nicotine addiction. Future research should consider whether the same complexities are evident for other addictive behaviors. 

## Figures and Tables

**Table 1:** Participant characteristics (N = 2768).

Characteristic	% (N)	M (SD)
COVID-19 diagnosis (%/N yes)	11.6% (321)	
Age		
21–30	21.3% (589)	
31–40	41.5% (1150)	
41–50	21.9% (605)	
51–59	15.3% (424)	
Gender		
Man	54.0% (1495)	
Woman	45.5% (1259)	
Transgender or gender non-conforming	0.5% (14)	
Race		
Asian	4.2% (116)	
Black or African American	20.6% (570)	
White/Caucasian	68.3% (1890)	
Multiracial	3.5% (97)	
Other race (i.e., Pacific Islander/Native Hawaiian, American Indian/Alaska Native, other)	3.4% (95)	
Ethnicity		
Hispanic/Latinx	13.4% (372)	
Non-Hispanic/Latinx	86.6% (2396)	
Job loss during COVID (%/N yes)	21.3% (589)	
Income loss due to COVID (%/N yes)	40.1% (1111)	
Weeks since first survey date		6.66 (4.06)
Stress during COVID		2.14 (0.63)
Heaviness of Smoking Index		2.53 (1.31)
Self-efficacy for quitting smoking		5.45 (2.77)
Desire to quit to quit smoking		6.31 (2.69)
Personal susceptibility (*n* = 2418) ^a^		
Low (1–2)	60.9% (1472)	
Moderate (3)	25.6% (619)	
High (4–5)	13.5% (327)	
Personal seriousness (*n* = 2418) ^a^		
Low (1–2)	26.3% (637)	
Moderate (3)	25.6% (619)	
High (4–5)	48.1% (1162)	
General susceptibility (%/N high)	53.8% (1489)	
General seriousness (%/N high)	62.3% (1724)	
Increased smoking (%/N yes)	23.7% (655)	
Decreased smoking (%/N yes)	11.3% (314)	

^a^ Only participants without a COVID-19 diagnosis reported personal susceptibility and seriousness. Participants with valid data for both personal susceptibility and personal seriousness are included here.

**Table 2:** Crosstabulations of personal and general seriousness and susceptibility.

**General susceptibility X general seriousness ^a^**					
		*General seriousness*			
		Decreases/no change		Increases	
General susceptibility	Decreases/no change	868 (31.4%)		411 (14.8%)	*χ*^2^ (1) = 919.97 *p* < 0.001
	Increases	176 (6.4%)		1313 (47.4%)	
**Personal seriousness X personal susceptibility ^b^**					
		*Personal seriousness*			
Personal susceptibility		Low	Moderate	High	
	Low	549 (22.7%)	351 (14.5%)	572 (23.7%)	*χ*^2^ (4) = 318.86 *p* < 0.001
	Moderate	72 (3.0%)	221 (9.1%)	326 (13.5%)	
	High	16 (0.7%)	47 (1.9%)	264 (10.9%)	
**General susceptibility X personal susceptibility ^b^**					
		*Personal susceptibility*			
		Low	Moderate	High	
General susceptibility	Decreases/no change	848 (35.1%)	253 (10.5%)	103 (4.3%)	*χ*^2^ (2) = 99.45, *p* < 0.001
	Increases	624 (25.8%)	366 (15.1%)	224 (9.3%)	
**General seriousness X personal seriousness ^b^**					
		*Personal seriousness*			
General seriousness		Low	Moderate	High	
	Decreases/no change	382 (15.8%)	236 (9.8%)	334 (13.8%)	*χ*^2^ (2) = 168.60, *p* < 0.001
	Increases	255 (10.5%)	383 (15.8%)	828 (34.2%)	

^a^ N = 2768; ^b^ N = 2418.

**Table 3:** Changes in smoking and general COVID-related perceptions among adults who smoke daily in 30 U.S. cities (N = 2768).

Variable	Increased Smoking OR [95% CI]	Decreased Smoking OR [95% CI]
COVID-19 diagnosis (Ref: no COVID-19 diagnosis)	**2.38 (1.78, 3.18)**	**2.52 (1.78, 3.57)**
Age (Ref: 21–30)		
31–40	0.83 (0.65, 1.06)	**0.61 (0.44, 0.83)**
41–50	**0.67 (0.50, 0.88)**	**0.47 (0.33, 0.69)**
51–59	**0.39 (0.27, 0.57)**	**0.32 (0.17, 0.59)**
Gender: Man (Ref: womxn) ^a^	0.97 (0.80, 1.17)	**1.69 (1.26, 2.27)**
Race/ethnicity: POC ^b^ (Ref: Non-Hispanic white)	1.06 (0.87, 1.29)	0.86 (0.66, 1.14)
Job loss during the COVID-19 pandemic (Ref: no job loss)	1.18 (0.93, 1.49)	**1.50 (1.09, 2.07)**
Income loss (Ref: no income loss or unknown)	1.17 (0.96, 1.44)	1.12 (0.84, 1.49)
Time ^c^	0.99 (0.96, 1.01)	1.00 (0.96, 1.03)
Stress during COVID	**1.91 (1.61, 2.27)**	**1.75 (1.41, 2.17)**
Heaviness of Smoking Index	**1.28 (1.19, 1.39)**	**0.87 (0.78, 0.97)**
Self-efficacy	0.97 (0.93, 1.02)	**1.20 (1.12, 1.28)**
Desire to quit	**1.07 (1.03, 1.12)**	1.07 (1.00, 1.14) ^d^
High General susceptibility (Ref: unchanged/low)	**1.61 (1.25, 2.06)**	1.22 (0.83, 1.78)
High General seriousness (Ref: unchanged/low)	**0.76 (0.60, 0.98)**	0.73 (0.50, 1.06)

^a^ Womxn = female, transgender, or gender non-conforming; ^b^ POC = person of color (i.e., someone who identifies as Hispanic/Latinx and/or a race other than white); ^c^ Time was measured as weeks elapsed since first survey date (14 July 2020); ^d^ Despite a lower bound of 1.00 in the 95% confidence interval, desire to quit was not significantly associated with decreased smoking at *p* < 0.05. The lower bound was rounded up to 1.00.

**Table 4:** Changes in smoking and COVID-related perceptions among adults who smoke daily and have not had COVID-19 in 30 U.S. cities (N = 2418).

Variable	Increased Smoking OR [95% CI]	Decreased Smoking OR [95% CI]
Age (Ref: 21–30)		
31–40	0.78 (0.60, 1.02)	**0.63 (0.44, 0.90)**
41–50	**0.55 (0.40, 0.75)**	**0.37 (0.23, 0.60)**
51–59	**0.35 (0.24, 0.52)**	**0.33 (0.17, 0.63)**
Gender: Man ^a^ (Ref: womxn)	0.92 (0.75, 1.13)	**1.50 (1.07, 2.10)**
Race/ethnicity: POC ^b^ (Ref: non-Hispanic white)	1.15 (0.92, 1.44)	0.78 (0.56, 1.09)
Job loss during COVID-19 pandemic (Ref: no job loss)	1.28 (0.97, 1.68)	**2.12 (1.43, 3.14)**
Income loss (Ref: no income loss)	1.18 (0.94, 1.49)	0.88 (0.62, 1.26)
Time ^c^	0.98 (0.96, 1.01)	1.00 (0.96, 1.04)
Stress during COVID	**1.78 (1.44, 2.20)**	**1.84 (1.37, 2.46)**
Heaviness of Smoking Index	**1.33 (1.23, 1.45)**	**0.84 (0.74, 0.95)**
Self-efficacy	**0.95 (0.91, 1.00) ^d^**	**1.15 (1.07, 1.24)**
Desire to quit	**1.06 (1.01, 1.11)**	1.06 (0.99, 1.14)
Personal susceptibility (Ref: low 1–2)		
Moderate (3)	1.05 (0.81, 1.36)	1.04 (0.67, 1.60)
High (4–5)	**1.77 (1.30, 2.42)**	**1.86 (1.18, 2.93)**
Personal seriousness (Ref: low 1–2)		
Moderate (3)	1.15 (0.84, 1.58)	1.05 (0.64, 1.72)
High (4–5)	**1.52 (1.14, 2.04)**	1.15 (0.73, 1.81)
High general susceptibility (Ref: moderate/low)	**1.45 (1.11, 1.90)**	1.36 (0.87, 2.13)
High general seriousness (Ref: moderate/low)	**0.71 (0.54, 0.93)**	**0.56 (0.36, 0.86)**

^a^ Womxn = female, transgender, or gender non-conforming; ^b^ POC = person of color (i.e., someone who identifies as Hispanic/Latinx and/or a race other than white); ^c^ Time was measured as weeks elapsed since first survey date (14 July 2020); ^d^ Despite an upper bound of 1.00 in the 95% confidence interval, self-efficacy was significantly negatively associated with increased smoking at *p* < 0.05. The upper bound was rounded up to 1.00. Bold text indicates *p* < 0.05.

## Data Availability

Data are available at the conclusion of the study upon reasonable request.

## Appendix A

**Table A1:** Participants and recruitment channels in each of 30 U.S. cities.

City	Recruitment Channels	*n* in Analytic Sample
Atlanta, GA	Qualtrics, Craigslist	95
Baltimore, MD	Qualtrics	100
Boston, MA	Qualtrics, Craigslist	89
Charlotte, NC	Qualtrics, Craigslist	97
Chicago, IL	Qualtrics	95
Cleveland, OH	Qualtrics, Craigslist	89
Dallas, TX	Qualtrics, Craigslist	98
Denver, CO	Qualtrics	91
Detroit, MI	Qualtrics, Craigslist	72
Fort Worth, TX	Qualtrics, Craigslist	85
Houston, TX	Qualtrics, Craigslist	96
Kansas City, MO	Qualtrics, Craigslist	97
Las Vegas, NV	Qualtrics	87
Los Angeles, CA	Qualtrics, Craigslist	99
Memphis, TN	Qualtrics, Craigslist	103
Miami, FL	Qualtrics, Craigslist	85
Minneapolis, MN	Qualtrics, Craigslist	65
New Orleans, LA	Qualtrics, Craigslist	97
New York, NY	Qualtrics, Craigslist	106
Oakland, CA	Qualtrics, Craigslist	86
Philadelphia, PA	Qualtrics	90
Phoenix, AZ	Qualtrics	97
Portland, OR	Qualtrics, Craigslist	101
Providence, RI	Qualtrics, Craigslist	95
Sacramento, CA	Qualtrics, Craigslist	84
San Antonio, TX	Qualtrics, Craigslist	92
San Diego, CA	Qualtrics, Craigslist	112
San Francisco, CA	Qualtrics, Craigslist	96
Seattle, WA	Qualtrics	92
Washington, D.C.	Qualtrics, Craigslist	77
